# Talent-Management in Nursing: Factors, Organisational Practices and Retention Indicators Among Portuguese Nurses—A Cross-Sectional Study

**DOI:** 10.3390/nursrep16090343

**Published:** 2026-09-19

**Authors:** Telma Quaresma, Maria Céu Marques, Liliana Mota

**Affiliations:** 1 CHRC—Comprehensive Health Research Centre, University of Évora, 7004-516 Évora, Portugal; telma.quaresma@ulsalg.min-saude.pt; 2 Department of Education, Innovation and Research, Algarve Local Health Unit—Hospital Unit of Portimão, 8500-338 Portimão, Portugal; mcmarques@uevora.pt; 3 São João de Deus School of Nursing, University of Évora, 7004-516 Évora, Portugal; 4 Portuguese Red Cross Northern Health School, 3720-126 Oliveira de Azeméis, Portugal; 5 RISE-Health, Faculty of Medicine, University of Porto, 4200-319 Porto, Portugal

**Keywords:** talent management, nurse retention, nursing workforce, human resource management, nursing leadership, workforce sustainability

## Abstract

**Introduction:** Talent management is increasingly relevant to nursing workforce sustainability, yet limited evidence has examined how nurses simultaneously perceive talent-related factors, organisational practices, and attraction and retention outcomes. **Objective:** The aim of this study was to examine the relationships between talent-management factors, organisational talent-management practices and perceived attraction and retention outcomes, while examining differences according to nurses’ sociodemographic and professional characteristics. **Methodology:** A multicentre, descriptive-correlational cross-sectional study was conducted with 1004 nurses from 29 public and private hospital organisations across Portugal. Talent management was assessed using the 87-item Talent Attitudes Questionnaire, comprising 12 subdimensions across factors, practices and organisational indicators. **Results**: Nurses reported favourable perceptions of talent-management factors (M = 3.34), particularly leadership competencies, but less favourable organisational practices (M = 3.02). Attraction (M = 2.27) and retention (M = 2.31) had the lowest scores, whereas perceived consequences of talent management were high (M = 3.49), indicating a discrepancy between talent recognition and perceived organisational capacity to attract and retain nurses. Attraction and retention were strongly correlated (r = 0.77), while organisational facilitators showed moderate associations with both outcomes. Sector-related differences were observed for turnover and inhibiting factors. **Discussion:** The findings suggest a potential talent-management translation gap, an interpretive, descriptive framing rather than a directly tested construct, whereby recognition of talent and its organisational consequences coexists with limited perceived capacity to attract and retain nurses. **Conclusions**: Nursing workforce sustainability requires translating talent recognition into organisational conditions that support professional development, attraction, and retention. These findings provide an integrated organisational perspective on talent management and nurse retention.

## 1. Introduction

Healthcare systems increasingly depend on their capacity not only to recruit nurses but also to develop, recognise and retain the professional expertise required to ensure the quality, continuity and sustainability of care. Persistent nursing shortages, workforce mobility and the loss of experienced professionals have therefore shifted attention from conventional recruitment strategies towards broader organisational approaches capable of supporting workforce sustainability. Within this context, talent management has gained increasing relevance as a strategic approach to identifying, developing and retaining professionals whose knowledge, competencies and potential contribute to organisational performance and sustainability [1]. This developmental perspective is particularly relevant to nursing. Although Benner’s model [2] was not developed as a talent-management framework, its conceptualisation of nursing expertise as progressively acquired through situated clinical experience provides an important theoretical lens for understanding the developmental dimension of professional talent [3]. From this perspective, expertise is not a fixed individual characteristic but the outcome of progressive learning, reflective practice and accumulated experience within specific clinical contexts. Identifying and developing professional competencies consequently becomes particularly important in healthcare organisations, where the continuity of qualified professionals is fundamental to the quality and resilience of health services [4]. Similarly, identifying emerging nurse leaders and investing in leadership competencies are important strategies for ensuring leadership continuity and strengthening organisational capacity [5,6].

However, professional talent does not develop independently of the organisational environment. The extent to which nurses can develop and apply their competencies is influenced by organisational structures, leadership, professional development opportunities, recognition and working conditions. People management therefore constitutes a strategic organisational function, and talent-management practices can support the identification, development and retention of professionals with the potential to contribute to organisational objectives [7,8].

In this respect, Chiavenato’s systemic approach to people management [9] complements Benner’s developmental perspective [2]. Whereas Benner provides a theoretical lens for understanding how expertise develops progressively within the individual through experience and professional practice, Chiavenato provides an organisational perspective for examining how human potential can be identified, developed and strategically managed. The integration of these perspectives allows for talent management in nursing to be considered simultaneously as an individual-developmental and an organisational phenomenon.

Within this integrated framework, healthcare organisations have a responsibility to create structural, cultural and relational conditions that enable professional potential to develop and be sustained. Succession planning, leadership development and the strategic allocation of human resources may contribute to preparing future generations of professionals and maintaining the expertise required to ensure quality of care and patient safety [10,11]. Organisational practices related to selection and competency development may also influence nurses’ commitment and professional satisfaction [12], while nursing leadership can act as an interface between organisational policies and clinical practice, contributing to professional environments that support institutional sustainability [13].

Talent in nursing can consequently be conceptualised as a multidimensional and relational construct encompassing clinical expertise, relational capabilities, ethical principles, leadership competencies and the capacity to make effective decisions in complex and unpredictable care environments [14]. Its expression results not only from education and accumulated professional experience but also from the interaction between continuous professional development and organisational contexts that provide opportunities, autonomy, recognition and appropriate working conditions. Talent management in nursing should therefore extend beyond identifying high-performing individuals to encompass the organisational processes through which professional capabilities are recognised, developed, supported and retained.

Despite increasing attention to nursing workforce sustainability, important gaps remain in understanding talent management within healthcare organisations. Research has frequently examined workforce outcomes and their determinants through specific constructs, including recruitment, professional development, leadership, turnover and retention. However, less attention has been given to examining, within the same nursing workforce, how nurses perceive the factors that characterise professional talent, the organisational practices intended to manage and develop that talent, and the organisational outcomes associated with its attraction and retention.

This distinction is particularly relevant because recognising professional talent and acknowledging its strategic importance do not necessarily mean that healthcare organisations are able to translate this recognition into effective organisational practices. A potential discrepancy may therefore exist between the recognition of talent, the organisational conditions created to support it, and the capacity of healthcare organisations to attract and retain professionals. Examining these dimensions simultaneously may provide a more comprehensive understanding of talent management than approaches that consider attraction, retention, professional development or turnover as isolated workforce phenomena.

In particular, the relationships between organisational talent-management practices and perceived attraction and retention outcomes warrant further examination. Organisational conditions that facilitate professional development and recognition may be associated with more favourable perceptions of attraction and retention, whereas organisational barriers may constrain the capacity to sustain professional talent. Understanding these relationships is therefore important for identifying where the transition from recognising professional talent to effectively managing and retaining it may be weakened within healthcare organisations. Building explicitly on this theoretical integration, three propositions guided the present study: (P1) consistent with Benner’s developmental perspective, individual talent-related factors are expected to reflect the progressive, experience-based acquisition of professional expertise; (P2) consistent with Chiavenato’s systemic approach, organisational talent-management practices, particularly facilitating conditions such as supportive leadership, recognition and development opportunities, are expected to show a stronger association with perceived attraction and retention than talent-related factors alone, because these practices operationalise the organisational mechanisms through which talent is sustained; and (P3) because organisations may recognise professional talent without necessarily translating that recognition into effective practices, a discrepancy may be observed between perceptions of talent-related factors and perceptions of organisational attraction and retention capacity. These propositions are examined empirically in the Results and Discussion.

Nurses’ sociodemographic and professional characteristics may also contribute to differences in how talent and organisational talent-management practices are perceived. Professional experience, career stage, academic qualifications, professional category and organisational context may shape expectations regarding professional development, leadership, recognition and retention. Considering these characteristics alongside talent-management factors, organisational practices and attraction and retention outcomes may therefore provide a more differentiated understanding of how talent management is experienced across the nursing workforce.

In Portugal, this issue is particularly relevant. The National Health Service (NHS) faces structural challenges in nursing workforce management, including staff shortages, professional dissatisfaction, burnout and the emigration of qualified professionals seeking more favourable working and remuneration conditions [15]. These challenges make the capacity to attract and retain nursing professionals particularly important for workforce sustainability. Nevertheless, research specifically examining talent management within the Portuguese nursing clinical context remains limited.

The Portuguese context therefore provides an opportunity to examine a broader organisational question that extends beyond a geographically specific workforce problem: whether the recognition and development of nursing talent are accompanied by organisational practices and conditions perceived as capable of supporting talent attraction and retention. Investigating these relationships may contribute to understanding how talent-management strategies are translated into organisational conditions associated with workforce sustainability.

Against this theoretical and empirical background, the present study integrates Benner’s competency development model [2] with Chiavenato’s systemic approach to people management [9] to examine talent management from both individual-developmental and organisational perspectives. Using the Talent Attitudes Questionnaire (QAF-T) [16], the study simultaneously considers talent-management factors, organisational talent-management practices and organisational indicators, thereby enabling an integrated examination of how the recognition of professional talent relates to organisational conditions associated with its attraction and retention.

Therefore, the overall aim of this study was to examine the relationships between talent-management factors, organisational talent-management practices and perceived attraction and retention outcomes, while examining differences according to nurses’ sociodemographic and professional characteristics. Specifically, the study aimed to characterise nurses’ perceptions of talent-management factors and organisational talent-management practices; assess perceived organisational indicators of talent attraction, retention, and turnover and the consequences of talent management; examine the relationships between talent-management factors, organisational practices, and attraction and retention outcomes; and explore whether these perceptions differed according to nurses’ sociodemographic and professional characteristics. By examining these dimensions simultaneously within a large, multicentre nursing sample, the study seeks to provide an integrated organisational perspective on the relationship between talent recognition, organisational talent-management practices and nursing workforce attraction and retention.

## 2. Materials and Methods

### 2.1. Study Design

A multicentre, quantitative, descriptive-correlational, cross-sectional study was conducted among nurses working in Portuguese healthcare organisations. The study followed the Strengthening the Reporting of Observational Studies in Epidemiology (STROBE) recommendations for cross-sectional observational studies [16] (Supplementary Table S1). This design enabled the characterisation of nurses’ perceptions of talent-management factors, organisational talent-management practices and organisational indicators, as well as the examination of associations between QAF-T scores and participants’ sociodemographic and professional characteristics.

### 2.2. Population and Sample

The target population comprised nurses working in public and private healthcare organisations in Portugal. A non-probability convenience sampling strategy was used, based on voluntary participation among nurses from the participating institutions. Participating institutions were recruited through institutional contacts established by the research team and were included following formal institutional and ethics approval.

Eligible participants were registered nurses actively working in public or private healthcare institutions in mainland Portugal or the autonomous regions and who voluntarily agreed to participate. Nursing students and professionals not belonging to the nursing profession were excluded. The final sample comprised 1004 nurses, from 29 public- and private-sector hospital organisations, distributed from the north to the south of Portuguese territory, including the autonomous regions. Participation was not evenly distributed across the 29 participating organisations, with the number of respondents per institution ranging from 5 to 169 (median = 34), consistent with a non-stratified, non-proportionally allocated recruitment design and variable participation across institutions.

No a priori sample size calculation was performed because recruitment was based on voluntary participation within the participating institutions. Given the non-probability sampling strategy, the sample should not be considered nationally representative, and this should be taken into account when interpreting the generalisability of the findings. Consequently, the findings and conclusions of this study should be interpreted as applying primarily to the participating organisations and sample, rather than being generalised to the Portuguese nursing workforce as a whole.

### 2.3. Variables

The study variables were organised into four categories:

Sociodemographic and professional variables (explanatory variables): age, gender, years of professional experience, academic qualifications, area of specialisation, professional category, area of practice, employment status, and sector of practice (public/private).

The main study variables comprised the 12 subdimensions of the Talent Attitudes Questionnaire (QAF-T) [17], organised into talent-management factors, talent-management practices and organisational talent-management indicators. Each subdimension was analysed according to its corresponding score.

The primary perceived organisational outcomes of interest were talent attraction (Q9) and talent retention (Q10). Turnover indicators (Q11) and perceived consequences of talent management (Q12) were examined as additional organisational talent-management indicators. Higher subdimension scores reflected greater endorsement of the respective talent-management indicators.

### 2.4. Data Collection Instrument

#### 2.4.1. Sociodemographic and Professional Data

The questionnaire included a sociodemographic and professional characterisation section, collecting information on: age, gender, sector of practice (public or private), academic qualifications, area of specialisation, professional category, area of clinical practice, and employment status. These data were collected simultaneously with the administration of the Talent Attitudes Questionnaire [17]. Internal consistency of the QAF-T subdimensions was assessed in the present sample using Cronbach’s alpha.

#### 2.4.2. Talent Attitudes Questionnaire

Talent management was assessed using the Talent Attitudes Questionnaire (QAF-T) [17], a self-report instrument comprising 87 items organised into three main domains and 12 subdimensions: talent-management factors, talent-management practices and organisational talent-management indicators (Table 1). Items are rated on a four-point Likert scale. Higher scores indicate greater endorsement of the construct assessed within each respective subdimension.

In the present study, with a sample of 1004 nurses, the internal consistency of the instrument was assessed using Cronbach’s alpha coefficient by subdimension and for the overall instrument. Subdimension coefficients ranged from α = 0.741 for inhibiting factors to α = 0.968 for facilitating factors, while the overall 87-item instrument yielded α = 0.938; all subdimensions therefore showed Cronbach’s alpha coefficients ≥ 0.70 [18]. These coefficients provide evidence of internal consistency only and should not be interpreted as evidence of the instrument’s structural validity. Confirmatory or exploratory factor analysis, factor loadings, convergent and discriminant validity, composite reliability, average variance extracted (AVE) and measurement invariance across groups (e.g., gender, professional category, academic qualification, and sector) were not assessed in the present sample. Given that the study makes comparisons across these groups, this represents an important limitation, and future research should formally test the 12-dimensional structure of the QAF-T—building on the original exploratory validation study [17]—including measurement invariance testing, before group comparisons are interpreted as reflecting a fully equivalent underlying structure across subgroups. Data were collected between September 2022 and March 2023 using an electronic questionnaire administered through Google Forms. Within participating institutions, the study invitation and electronic questionnaire link were disseminated to eligible nurses through institutional communication channels. Participation was voluntary, and no individual sampling frame or random selection of nurses was used. All questionnaire items were configured as mandatory in an electronic form; consequently, the analytical dataset contained no missing responses for the QAF-T items. The completion of the questionnaire took approximately 20–30 min.

Prior to data collection, formal written authorisation to use the Portuguese-language version of the QAF-T [17] was obtained from the instrument’s original author. To enable its administration at the participating institutions, requests for institutional authorisation were submitted to the Boards of the respective Hospital Centres, as well as to the competent Ethics Committees, with all procedures complying with the regulatory standards of each organisation.

To mitigate potential response and measurement biases, the questionnaire was administered anonymously and without supervision by nurse managers or line managers, and standardised instructions were provided to all participants. Nevertheless, the voluntary convenience sampling strategy may have introduced selection and self-selection bias.

### 2.5. Ethical Approval

The study was conducted in accordance with the Declaration of Helsinki and the Oviedo Convention and was approved by the Ethics Committee of the principal investigator’s institution (Opinion No. 22110). Ethical approval was also obtained from the participating institutions, in accordance with their respective institutional requirements. Participation was voluntary, and all participants provided informed consent before accessing the questionnaire. Anonymity and confidentiality were ensured throughout data collection, processing and analysis.

### 2.6. Data Analysis

Data processing and analysis were carried out using the software Statistical Package for the Social Sciences (SPSS), version 29.0.

In the descriptive analysis stage, absolute and relative frequencies were calculated for categorical variables, and measures of central tendency (mean) and dispersion (standard deviation, minimum and maximum) were calculated for continuous variables.

Distributional assumptions were assessed using the Kolmogorov–Smirnov and Shapiro–Wilk tests, complemented by an inspection of skewness and kurtosis. Given the large sample size, decisions regarding the use of parametric or non-parametric procedures were not based solely on significance tests of normality.

For the inferential analysis, the following statistical procedures were used, selected according to the verification of normality assumptions and the nature of the variables. Associations between approximately normally distributed continuous composite scores were examined using Pearson’s correlation coefficient (r), whereas Spearman’s rank correlation coefficient (ρ) was used for ordinal variables or continuous variables that did not adequately meet distributional assumptions.

Statistical significance was set at α = 0.05. Effect sizes were reported alongside inferential tests to support interpretation beyond statistical significance. For Mann–Whitney U tests, effect size was calculated as r = Z/√*N*; for Kruskal–Wallis tests, η^2^ was reported; and Pearson correlation coefficients are presented with 95% confidence intervals obtained using Fisher’s z transformation. Effect sizes were interpreted according to established reference criteria [18]. Associations between talent-management factors, organisational practices and the attraction and retention outcomes were examined using bivariate correlation coefficients; multivariable methods (e.g., multiple or hierarchical regression) that could control simultaneously for sociodemographic and professional covariates (age, professional experience, gender, academic qualification, professional category, sector and organisational context) were additionally applied in a supplementary analysis, reported in Section 3, using ordinary least squares regression with heteroscedasticity-consistent (HC3) standard errors. In addition, given the large number of statistical tests performed across QAF-T dimensions and sociodemographic/professional variables, the Benjamini–Hochberg false discovery rate (FDR) procedure was additionally applied within each demographic comparison to correct for multiple comparisons, and results are reported both before and after this correction in Section 3; with a sample of this size, very small associations can reach statistical significance, and such analyses—particularly those with small effect sizes—should therefore be interpreted with caution, with emphasis placed on effect-size magnitude and confidence intervals rather than statistical significance alone.

## 3. Results

Of the 1007 eligible participants, three declined participation, resulting in a final analytical sample of 1004 nurses from 29 public and private hospital organisations across Portugal. The sociodemographic and professional characteristics of the sample are presented in Table 2.

The sample was predominantly female (81.9%) and employed in the public sector (90.8%), with most participants working in direct care (76.9%). Participants had a mean age of 42.58 years (SD = 9.76) and a mean professional experience of 19.44 years (SD = 9.97). Detailed sociodemographic and professional characteristics are presented in Table 2 and Table 3.

Mean scores for the dimensions assessed by the QAF-T ranged between 2.27 and 3.60, on a response scale from 1 (strongly disagree) to 4 (strongly agree). Descriptive statistics, together with skewness and kurtosis indicators for the three dimensions and their respective items, are presented in Table 4.

At the composite-domain level, talent-management factors showed the highest mean score (M = 3.34, SD = 0.37), followed by talent-management practices (M = 3.02, SD = 0.46) and talent-management indicators (M = 2.88, SD = 0.35). At the subdimension level, leader competencies showed the highest score (M = 3.60, SD = 0.46), whereas attraction (M = 2.27, SD = 0.79) and retention (M = 2.31, SD = 0.76) showed the lowest scores. In contrast, perceived consequences of talent management were highly rated (M = 3.49, SD = 0.50). This descriptive pattern indicates higher scores for the recognition of talent-related factors and consequences than for perceived organisational attraction and retention.

Differences in QAF-T scores were analysed according to participants’ sociodemographic and professional variables, with the aim of identifying any response patterns associated with different subgroups of the sample. The normality of the distribution of scores was previously assessed using the Kolmogorov–Smirnov and Shapiro–Wilk tests, the results of which determined the selection of non-parametric statistical tests for the subsequent analyses. To analyse differences in QAF-T scores according to participants’ gender, the Mann–Whitney U test was applied, given the non-fulfilment of normality assumptions. The results for the two dimensions in which statistically significant differences were identified are presented in Table 5.

Statistically significant gender differences were identified for concept of talent (Q1; U = 64,652.5, Z = −2.882, *p* = 0.004) and motivating factors (Q6; U = 66,062.5, Z = −2.488, *p* = 0.013). Male participants showed higher mean ranks for Q1, whereas female participants showed higher mean ranks for Q6. No statistically significant gender differences were observed for the remaining QAF-T subdimensions.

The Pearson correlation matrix between the twelve QAF-T dimensions is presented in Table 6. Most correlations were statistically significant (*p* < 0.05 or *p* < 0.01), with magnitudes ranging from weak to moderate to strong. Attraction (Q9) and retention (Q10) showed the strongest correlation observed across the QAF-T subdimensions (r = 0.77, *p* < 0.01). Facilitating factors (Q7) were moderately correlated with attraction (r = 0.51, *p* < 0.01) and retention (r = 0.47, *p* < 0.01). These associations were substantially stronger than most other correlations involving attraction and retention.

Table 7 presents the Pearson correlations between the continuous variables (age, length of professional practice, and time in the current organisation) and the QAF-T dimensions that showed statistically significant correlations with these variables, namely, young talent competencies (Q3), facilitating factors (Q7) and consequences of talent management (Q12).

The results show weak, but statistically significant, negative correlations between age and the valuing of the young-talent construct (r = −0.09; *p* < 0.01), as well as between length of professional practice (r = −0.08; *p* < 0.05) and time in the current organisation (r = −0.09; *p* < 0.01) and this same dimension. Conversely, weak positive correlations were found between these three temporal variables and the perception of facilitating organisational factors (Q7). Age, length of professional practice and time in the current organisation showed statistically significant but very weak correlations with young talent competencies (|r| ≤ 0.09) and facilitating factors (|r| ≤ 0.08). Given their small magnitude, these associations should be interpreted cautiously despite statistical significance.

To further examine these associations while accounting for potential confounding, multivariable linear regression models were additionally estimated for the three QAF-T domain scores and for the attraction (Q9) and retention (Q10) indicators, entering age, professional experience, time in the current organisation, gender, academic qualification, professional category and sector as covariates, with heteroscedasticity-consistent (HC3) standard errors. Professional category showed the most consistent independent association across outcomes, with specialist nurses and nurse managers scoring significantly higher than generalist nurses on all five outcomes (B ranging from 0.10 to 0.32; all *p* ≤ 0.02), net of the other covariates. Gender remained independently associated only with talent-management practices (B = 0.11, *p* = 0.007), with women scoring higher after adjustment. Academic qualification showed a mixed pattern, with nurses holding a Master’s degree scoring significantly lower on attraction and retention than those with a Licenciatura (B = −0.19 and B = −0.17, respectively; both *p* ≤ 0.01). Age, professional experience and time in the current organisation were not independently associated with any outcome once professional category was accounted for, except for a small negative association between age and talent-management factors (B = −0.012, *p* = 0.014); age and length of professional experience were collinear (variance inflation factor ≈ 14–16), which should be considered when interpreting their individual coefficients. The explanatory power of these models was modest (adjusted R^2^ between 0.014 and 0.068), indicating that most of the variance in perceived talent-management factors, practices and indicators remains unexplained by the sociodemographic and professional variables measured in this study. Complementary models for turnover indicators (Q11) and inhibiting factors (Q8) showed that the sector-related difference remained significant and became more pronounced for turnover indicators (B = −0.22, *p* ≤ 0.001, higher in the public sector) but was no longer statistically significant for inhibiting factors (B = −0.10, *p* = 0.078) once the other covariates were taken into account.

Given the number of significance tests performed, the Benjamini–Hochberg false discovery rate (FDR) procedure was applied within each sociodemographic/professional comparison (15 tests per comparison: 12 subdimensions plus 3 domain scores). Of the 35 group comparisons that reached conventional significance (*p* < 0.05) across gender, sector, professional category, academic qualification and employment-status comparisons, 28 remained significant after FDR correction and 24 after the more conservative within-family Bonferroni correction. Findings for professional category are the most robust, with 12 of the 15 comparisons surviving FDR correction, consistent with the multivariable results above. By contrast, the previously reported gender differences in concept of talent (Q1; raw *p* = 0.004) and motivating factors (Q6; raw *p* = 0.013) did not survive correction for multiple comparisons (FDR-adjusted *p* = 0.059 and *p* = 0.096, respectively) and should therefore be interpreted as exploratory rather than confirmed findings. Effect sizes for all statistically significant comparisons remained small (r ≤ 0.12 for Mann–Whitney comparisons; η^2^ ≤ 0.06 for Kruskal–Wallis comparisons), reinforcing the recommendation that statistical significance be interpreted alongside effect-size magnitude, particularly in a sample of this size.

## 4. Discussion

This study examined the relationships between talent-management factors, organisational talent-management practices, and perceived attraction and retention outcomes among nurses working in Portuguese healthcare organisations. Three main findings emerged. First, nurses reported favourable perceptions of talent-related factors, particularly leadership competencies, while attraction and retention received substantially lower scores. Second, organisational facilitating factors showed the strongest associations with both attraction and retention. Third, attraction and retention were strongly interrelated, suggesting that these outcomes represent closely connected components of nurses’ perceptions of organisational talent management. Together, these findings indicate a potential gap between recognising the strategic importance of nursing talent and translating that recognition into organisational conditions perceived as capable of attracting and retaining professionals.

The results relating to the talent-management factors dimension (3.34 ± 0.37) reveal an overall positive and consistent perception, with particular emphasis on the items leader competencies (Q4; M = 3.60) and current challenges (Q5; M = 3.46). These values indicate that nurses identify leadership and challenge orientation as central attributes of talent in the clinical context, in line with contemporary conceptualisations that describe talent as a combination of input (high potential/excellent skills) and output (excellent performance and value creation) [19].

This orientation echoes the distinction proposed by Gallardo-Gallardo et al. [20] between a talent-as-object perspective (a set of measurable attributes) and a talent-as-subject perspective (a category of exceptional person) [21,22]. The results suggest that Portuguese nurses operate according to an integrative perspective, valuing technical competence (Q2; M = 3.40) simultaneously with leadership qualities and an orientation towards excellence, consistent with the hybrid approach identified in the more recent literature [23].

The lower valuation of the young talent item (Q3; M = 3.16), associated with negative correlations with age (r = −0.09, *p* < 0.01) and length of professional practice (r = −0.08, *p* < 0.05), suggests, albeit on the basis of a very small effect size that warrants cautious interpretation, that more experienced nurses may place somewhat less value on talent associated with youth. This trend reflects intergenerational dynamics widely documented in the literature, according to which professional experience tends to shift the emphasis from latent potential to demonstrated competence [24], with direct implications for the intergenerational management of talent in nursing, namely for the balance between attracting new professionals and retaining more experienced ones.

The significant differences by professional category across all subdimensions, with specialist and manager nurses consistently scoring higher than generalist nurses, are theoretically consistent with Benner’s model [2], which frames professional development as a progressive, situated process: over the course of the journey from novice to expert, the professional accumulates technical competencies and develops a greater capacity to read organisational contexts. This trajectory appears to be accompanied by a growing awareness of what constitutes talent in nursing, with direct implications for the design of talent-management programmes differentiated by career level, a gap identified in the literature as a priority for future research. The significant differences by academic qualifications (H = 16.32, *p* < 0.001 for Q1; H = 17.22, *p* < 0.001 for Q4) likewise show that a higher academic qualification was associated with a more elaborate awareness of what constitutes organisational talent, with professionals holding a Master’s or Doctoral degree showing the highest scores on the talent-management factors and indicators.

The talent-management practices dimension (3.02 ± 0.46) shows an intermediate score that, at the item level, reveals significant heterogeneity. The motivation item (Q6; M = 2.92) is positioned moderately, while the facilitators item (Q7; 2.67 ± 0.91) simultaneously records the lowest mean and the highest standard deviation of the entire instrument—a combination that is clinically and organisationally relevant, suggesting that the perception of organisational support (conditions, resources and institutional support for talent development) is extremely heterogeneous across professionals, services and organisations.

The international literature on talent management in the public sector systematically identifies structural constraints, bureaucratic rigidity, budgetary limitations and the absence of formalised recognition mechanisms as obstacles to the effective implementation of these practices [25,26]. Thunnissen and Buttiens [27], in Flemish and Dutch public organisations, documented analogous contextual factors. In the Portuguese NHS, these constraints are compounded by budgetary containment policies that have limited external recruitment and contributed to the ageing of the workforce. It is important to contextualise these results in light of the structural reorganisation of the NHS initiated on 1 January 2024, with the extension of the Local Health Units (ULSs) across the entire national territory (31 new ULSs, integrating hospital and primary care under single management). This reform, which took place after the data collection period (from September 2022 to March 2023), may alter the organisational determinants of talent management in the public sector, and future studies should therefore examine its impact on perceptions of attraction, retention and turnover. In the meantime, the results of the present study provide quantitative evidence of the perceived magnitude of these constraints in a sizeable and geographically diverse sample.

The analysis by gender revealed statistically significant differences in Q6 (motivation; U = 66,062.5; Z = −2.488; *p* = 0.013), with higher mean ranks among women, consistent with research pointing to a greater valuing by women of intrinsic motivation, interpersonal relationships and the sense of mission associated with clinical practice [28]. The greater valuation of individual talent among men (Q1; *p* = 0.004) may be related to different norms of professional self-presentation within a historically feminised context, a topic that warrants further qualitative investigation. However, neither of these gender differences (Q1 or Q6) remained statistically significant after correction for multiple comparisons (Benjamini–Hochberg FDR-adjusted *p* = 0.059 and *p* = 0.096, respectively; see Section 3) and should therefore be regarded as exploratory rather than confirmed findings pending replication.

The results for the talent-management indicators dimension reveal the most concerning pattern in the study: the attraction (Q9; M = 2.27) and retention (Q10; M = 2.31) items show the lowest means of the entire instrument, below the scale’s theoretical midpoint (2.5). By contrast, the consequences of talent management (Q12; M = 3.49) and turnover (Q11; M = 3.33) items are among the highest. This paradoxical pattern—high awareness of the negative consequences of talent loss, combined with an unfavourable perception of the effectiveness of attraction and retention practices—constitutes the most substantive finding of this study.

This discrepancy is directly mirrored in the international literature and is identified across different geographical and organisational contexts as one of the most recurrent manifestations of talent management in the public health sector [29], frequently termed the talent-management gap: the gap between the strategic recognition of the importance of talent and its effective translation into sustained organisational practices [30]. In the Egyptian context, most nurses likewise showed low perceptions of talent management [31]; in the South African context, talent management in the public sector requires improvements in the implementation of practices, particularly in terms of active management involvement [32]. In the United Kingdom, Fisher et al. [33] showed, in a qualitative study within an NHS Trust, that talent-management initiatives can positively influence nurses’ retention intentions by creating support networks and organisational social capital [34], the perceived absence of which within the Portuguese NHS may be associated with the low values obtained for the attraction and retention indicators, although this interpretation remains speculative given the cross-sectional design. Taken together, these data from such distinct contexts as Portugal, South Africa and the United Kingdom converge on a common conclusion: recognising the problem does not automatically generate organisational response capacity, a dissociation that is particularly acute in public health systems with severe structural constraints. It should be noted that in the present study, the “talent-management translation gap” is proposed as a descriptive, interpretive framework, inferred from the observed difference between mean scores for talent-related factors/consequences and mean scores for attraction and retention, rather than a directly measured or statistically tested latent construct; these dimensions differ in content and number of items and are not necessarily directly comparable. Future studies should aim to operationalise and statistically test this gap explicitly, for example through a formally specified structural or difference-score model.

This reading is reinforced by the highly significant difference in turnover indicators (Q11) by sector (U = 32,125.5; Z = −3.734; *p* < 0.001), with higher scores in the public sector, and by the significant difference in inhibiting factors (Q8) by sector (U = 36,341.5; Z = −2.126; *p* = 0.033), also higher in the public sector. Kravariti and Johnston [25], in their critical review of talent management in the public sector, identify bureaucracy, budgetary limitations and the absence of formalised merit-recognition mechanisms as the main systemic obstacles to talent retention, a convergence of findings across Portugal, Turkey, South Africa, Egypt and the United Kingdom that points to a structural pattern that cuts across cultural contexts and the maturity of health systems. This asymmetry is further consistent with the literature acknowledging that in the public sector, organisational objectives are more diffuse and harder to measure, making the identification and recognition of talent more difficult [35]. The absence of significant differences between sectors in most of the remaining dimensions suggests, however, that these differences are operationally related to the structural conditions of implementation rather than conceptual: professionals in both sectors share a similar view of what talent is and of its organisational implications. This sector-related difference in turnover indicators remained robust, and its magnitude even increased in a multivariable model adjusting for age, professional experience, gender, academic qualification and professional category (B = −0.22, *p* ≤ 0.001), reinforcing the interpretation of a structural rather than purely compositional effect. By contrast, the sector-related difference in inhibiting factors (Q8) was attenuated and no longer statistically significant once these covariates were taken into account (B = −0.10, *p* = 0.078), nor after correction for multiple comparisons; this latter finding should accordingly be interpreted with greater caution than initially suggested by the bivariate analysis.

A particularly relevant finding was the moderate association of facilitating factors (Q7) with both attraction (Q9; r = 0.51) and retention (Q10; r = 0.47). Among the talent-management factors and practices examined, organisational facilitators showed the strongest relationships with these two outcomes. This suggests that nurses’ perceptions of attraction and retention are more closely associated with the organisational conditions that enable talent management than with the recognition of talent-related competencies alone. Although the cross-sectional design precludes causal inference, this pattern reinforces the relevance of organisational environments that provide opportunities for professional development, recognition, supportive leadership and conditions conducive to professional growth. The strong association observed between attraction (Q9) and retention (Q10) (r = 0.77; *p* < 0.001) further reinforces this organisational perspective. Although attraction and retention represent conceptually distinct organisational outcomes, their strong relationship suggests that they may share underlying organisational determinants. From a management perspective, this finding supports addressing attraction and retention as interconnected components of workforce strategy rather than as independent challenges.

The findings have relevant implications for organisational management and nursing workforce policy. Firstly, the low perceived effectiveness of attraction and retention processes, combined with the high awareness of their consequences, calls for systemic responses that go beyond isolated recruitment practices and address the structural conditions of work, career progression, merit recognition and work–life balance [36]. The recent literature suggests that psychological safety is a critical retention factor in front-line healthcare contexts, which invites exploration of whether the low perceived effectiveness of organisational facilitators (Q7; M = 2.67) may be associated with the absence of psychologically safe environments within NHS organisations, a hypothesis that warrants specific future investigation [37].

Secondly, the heterogeneity of perceptions regarding organisational facilitators (Q7) suggests that talent-management interventions should be contextualised and differentiated at the level of services and organisations, rather than standardised system-wide, a perspective reinforced by recent research in Turkish hospital contexts, where customisation of practices at the service level is identified as a determinant of intervention effectiveness [38].

Thirdly, the hierarchical gradient observed in the differences by professional category points to the need for talent-management strategies differentiated by career levels, recognising the distinct needs, motivations and expectations of nurses, specialist nurses and nurse managers—all the more important given that professionals with higher levels of specialisation and academic training are, as a rule, the most vulnerable to leaving for contexts with better working and pay conditions [22], a vulnerability particularly documented among specialist nurses and those in management roles, for whom identification and succession planning constitute critical retention practices [6].

Sector-related differences were observed for turnover and inhibiting factors, with higher scores among nurses working in public organisations. These findings may reflect differences in organisational structures and employment contexts; however, they should be interpreted cautiously given the unequal representation of public- and private-sector participants. Further comparative studies with more balanced sectoral samples are needed to determine whether these differences are consistently reproduced.

### Limitations

Several limitations should be considered. First, the cross-sectional design precludes causal inference regarding the relationships between talent-management practices and attraction or retention outcomes.

Second, convenience sampling may have introduced self-selection bias and limits the representativeness of the sample. Participation was also unevenly distributed across the 29 participating organisations (5–169 nurses per institution), which may further constrain generalisability.

Third, the predominance of nurses from public-sector organisations limits direct comparisons between public and private settings.

Fourth, all variables were self-reported, potentially introducing common-method and social-desirability biases. Finally, attraction and retention were assessed as perceived organisational indicators rather than objective outcomes, such as actual recruitment rates, turnover, or longitudinal retention data.

Fifth, the psychometric evaluation of the QAF-T in this sample was limited to internal consistency (Cronbach’s alpha); structural validity procedures (e.g., confirmatory factor analysis, convergent and discriminant validity, and measurement invariance) were not performed and should be addressed in future validation studies, particularly given the group comparisons reported here.

Sixth, although supplementary multivariable regression models and a Benjamini–Hochberg false discovery rate correction for multiple comparisons were subsequently applied and are reported in Section 3, these analyses remain exploratory: the models’ explanatory power was modest (adjusted R^2^ ≤ 0.068), some covariates were collinear (notably age and length of professional experience), and residual confounding by unmeasured organisational-level variables (e.g., staffing ratios, leadership style, and local human-resource practices) cannot be excluded; associations with small effect sizes should still be interpreted cautiously. Finally, the “talent-management translation gap” proposed in this study is an interpretive framing derived from between-dimension score differences rather than a directly tested construct, and future research should seek to operationalise and statistically test it.

## 5. Conclusions

This study provides an integrated perspective on talent management in nursing by examining talent-related factors, organisational practices, and perceived attraction and retention outcomes within the same workforce. Nurses reported favourable perceptions of talent-related factors and recognised the organisational consequences of talent management, while perceptions of organisational capacity to attract and retain talent were considerably less favourable. This discrepancy highlights a potential talent-management translation gap between recognising the strategic value of nursing talent and translating that recognition into organisational conditions perceived as capable of attracting and retaining professionals. This proposed translation gap is an interpretive, descriptive framing of the observed pattern rather than a construct that was directly measured or statistically tested in this study, and it should be formally operationalised and tested in future research.

Organisational facilitating factors showed the strongest associations with both attraction and retention, while these two outcomes were themselves strongly interrelated. These findings suggest that attraction and retention should not be approached as isolated workforce challenges but as interconnected components of a broader organisational talent-management strategy. Healthcare organisations may therefore benefit from strengthening conditions that support professional development, recognition, supportive leadership, career progression, and opportunities for nurses to develop and apply their competencies.

Although the cross-sectional design does not allow for causal relationships to be established, the findings extend retention-focused approaches by showing how talent recognition, organisational practices, and attraction and retention coexist within the same nursing workforce. Future longitudinal and intervention studies should examine whether strengthening organisational facilitators translates into measurable improvements in nurse attraction and retention. This perspective may contribute to the development of more integrated and sustainable talent-management strategies in nursing, both in Portugal and in comparable healthcare contexts.

## Figures and Tables

**Table 1:** Dimensional structure of the Talent Attitudes Questionnaire.

Main Domain	Subdimension	Item Statement	No. of Items
**Talent-Management Factors**	1. Concept of talent	In my opinion, the talented employee is…	7
	2. Talent competencies	I consider that the talented employee is the one who has the competency to…	9
	3. Young talent competencies	I understand that young talent demonstrates…	5
	4. Leader competencies	I consider that the leader has the competency to…	9
**Talent-Management Practices**	5. Current challenges	I think the great challenge organisations face today is…	6
	6. Motivating factors	In my opinion, what motivates the implementation of Talent Management practices in my organisation is…	6
	7. Facilitating factors	I feel that what facilitates the implementation of Talent Management practices in my organisation is…	7
**Talent-Management Indicators**	8. Inhibiting factors	In my organisation, I regard as an inhibiting factor of Talent Management…	7
	9. Attraction indicators	I consider that what makes my organisation attractive to talent is…	8
	10. Retention indicators	I understand that what contributes to talent retention in my organisation is…	9
	11. Turnover indicators	In my organisation, I regard as an indicator of *turnover* the voluntary departure of staff.	7
	12. Consequences of Talent Management	I consider that Talent Management practices have implications at the level of…	7

Note: Adapted from Ramalho (2011) [17].

**Table 2:** Sociodemographic and professional characterisation of the sample (N = 1004).

Variable	Category	n	%
Gender	Female	822	81.9
	Male	182	18.1
Sector	Public	912	90.8
	Private	92	9.2
Professional category	Nurse	609	60.6
	Specialist nurse	337	33.6
	Nurse manager	58	5.8
Current role	Direct care provision	772	76.9
	Management	68	6.8
	Both	164	16.3
Employment status	Public-sector employment contract (open ended)	378	37.6
	Individual employment contract—open ended (CITT)	568	56.6
	Fixed-term individual employment contract (CIT-TC)	24	2.4
	Other	34	3.4
OE Regional section	South	491	48.9
	North	255	25.4
	Centre	223	22.2
	Madeira	34	3.4
	Azores	1	0.1

Note: OE = Order of Nurses (Ordem dos Enfermeiros); CITT = open-ended individual employment contract; CIT-TC = fixed-term individual employment contract.

**Table 3:** Descriptive statistics for the continuous variables of the sample (N = 1004).

Variable	Min.	Max.	M	SD
Age (years)	22	67	42.58	9.76
Distance to workplace (km)	0.0	83.0	11.97	12.17
Length of professional practice (years)	0.0	44.0	19.44	9.97
Time in current organisation (years)	0.0	43.0	16.53	10.51

Note: M = Mean; SD = Standard deviation.

**Table 4:** Means, standard deviations and distribution indicators for the QAF-T dimensions and items (N = 1004).

Dimension/Item	Min.	Max.	M	SD	Skew./Kurt.
***Talent-Management Factors (3.34 ± 0.37)***					
Q1—Concept of talent	1.00	4.00	3.21	0.43	−0.18/−0.01
Q2—Talent competencies	1.33	4.00	3.40	0.43	−0.56/−0.10
Q3—Young talent competencies	1.20	4.00	3.16	0.47	−0.16/−0.18
Q4—Leader competencies	1.00	4.00	3.60	0.46	−1.49/3.49
Q5—Current challenges	1.00	4.00	3.46	0.44	−0.73/0.83
***Talent-Management Practices (3.02 ± 0.46)***					
Q6—Motivating factors	1.00	4.00	2.92	0.53	−0.46/0.97
Q7—Facilitating factors	1.00	4.00	2.67	0.91	−0.23/−1.03
Q8—Inhibiting factors	1.00	4.00	2.98	0.49	−0.20/0.47
***Talent-Management Indicators (2.88 ± 0.35)***					
Q9—Attraction indicators	1.00	4.00	2.27	0.79	0.44/−0.31
Q10—Retention indicators	1.00	4.00	2.31	0.76	0.27/−0.40
Q11—Turnover indicators	1.00	4.00	3.33	0.54	−0.86/1.23
Q12—Consequences of talent management	1.00	4.00	3.49	0.50	−0.85/1.38

Note: M = Mean; SD = Standard deviation; Skew. = Skewness; Kurt. = Kurtosis. Response scale from 1 (strongly disagree) to 4 (strongly agree). Values in parentheses correspond to the means and standard deviations of the composite dimensions.

**Table 5:** Differences in QAF-T scores by gender—dimensions with statistically significant differences (Mann–Whitney U test; N = 1004).

Dimension	Mean Rank (Female)	Mean Rank (Male)	U	Z	*p*	Effect Size r
Q1—Concept of talent	490.15	558.27	64,652.5	−2.882	0.004	0.091
Q6—Motivating factors	513.13	454.48	66,062.5	−2.488	0.013	0.079

Note: *p* < 0.05. Effect size calculated as r = |Z|/√N; both effects were small.

**Table 6:** Pearson correlations between the QAF-T dimensions (N = 1004).

	Q1	Q2	Q3	Q4	Q5	Q6	Q7	Q8	Q9	Q10	Q11	Q12
**Q1**	—	0.67 **	0.53 **	0.47 **	0.28 **	0.27 **	0.12 **	0.10 **	0.08 *	0.08 *	0.10 **	0.30 **
**Q2**		—	0.65 **	0.57 **	0.31 **	0.29 **	0.14 **	0.14 **	0.12 **	0.12 **	0.13 **	0.37 **
**Q3**			—	0.55 **	0.29 **	0.28 **	0.13 **	0.11 **	0.11 **	0.11 **	0.11 **	0.27 **
**Q4**				—	0.39 **	0.28 **	0.13 **	0.16 **	0.06	0.09 **	0.12 **	0.37 **
**Q5**					—	0.35 **	0.08 *	0.23 **	−0.03	−0.06	0.19 **	0.35 **
**Q6**						—	0.40 **	0.14 **	0.26 **	0.26 **	0.08 **	0.14 **
**Q7**							—	0.06	0.51 **	0.47 **	−0.03	0.05
**Q8**								—	−0.06 *	−0.08 *	0.36 **	0.23 **
**Q9**									—	0.77 **	−0.14 **	−0.02
**Q10**										—	−0.09 **	0.01
**Q11**											—	0.33 **
**Q12**												—

Note: * *p* < 0.05; ** *p* < 0.01.

**Table 7:** Pearson correlations between continuous variables and selected QAF-T dimensions (N = 1004).

Variable	Q3—Young Talent	Q7—Facilitators	Q12—Consequence
Age	−0.09 **	0.08 *	−0.06 *
Length of professional practice	−0.08 *	0.06 *	−0.05
Time in current organisation	−0.09 **	0.06 *	−0.06

Note: * *p* < 0.05; ** *p* < 0.01. Only dimensions with statistically significant correlations with the temporal variables are shown.

## Data Availability

The data presented in this study are available on reasonable request from the corresponding author due to privacy and ethical restrictions.

## Supplementary Materials

The following supporting information can be downloaded at: https://www.mdpi.com/article/10.3390/nursrep16090343/s1, Table S1: STROBE Statement—Checklist with Manuscript Page References.

## Public Involvement Statement

No members of the public were involved in the design, conduct, or reporting of this research beyond their participation as study respondents.

## Guidelines and Standards Statement

This manuscript was drafted in accordance with the STROBE (Strengthening the Reporting of Observational Studies in Epidemiology) reporting guidelines for observational research.
